# BMC International Health and Human Rights Reviewer Acknowledgement 2014

**DOI:** 10.1186/s12914-015-0044-0

**Published:** 2015-03-13

**Authors:** Giulia Mangiameli

**Affiliations:** BioMed Central, Floor 6, 236 Gray’s Inn Road, London, WC1X 8HB UK

## Abstract

The editors of BMC International health and human rights would like to thank all our reviewers who have contributed to the journal in Volume 14 (2014).

**Seye Abimbola**

Nigeria

**Elizabeth Abu-Haydar**

USA

**Philip Adongo**

Ghana

**Vishal Aggarwal**

UK

**Kingsley Agho**

Australia

**Mohamed Azmi Ahmad Hassali**

Malaysia

**Ameena Ahmed**

USA

**Moses Aikins**

Ghana

**George Annas**

USA

**Jennifer Anyanti**

Nigeria

**Odwa Atari**

Canada

**Chamila Thushari Attanapola**

Norway

**Edwinah Atusingwize**

Uganda

**Jenita Baruah**

India

**Gajananda Bhandari**

Nepal

**Ilse Blignault**

Australia

**Marleen Bosmans**

Belgium

**Stine Hellum Braathen**

Norway

**Robert Brooks**

Australia

**Garrett Brown**

UK

**Justine Bukenya**

Uganda

**Alasdair Cochrane**

UK

**Claudia Cappa**

USA

**Arachu Castro**

USA

**Rose Chapman**

Australia

**Jeremiah Chikovore**

South Africa

**Cari Clark**

United States Minor Outlying Islands

**Jocalyn Clark**

Bangladesh

**Todd Coleman**

Canada

**Anne Lia Cremers**

Netherlands

**Joanne Csete**

USA

**Lucia D'Ambruoso**

UK

**Eugene Kofuor Maafo Darteh**

Ghana

**Bregje De Kok**

UK

**Richard Elliott**

Canada

**Charli Eriksson**

Sweden

**Kathryn Falb**

USA

**Allison Friedman**

USA

**Eric Friedman**

USA

**Ines Fronteira**

Portugal

**Ana Gama**

Portugal

**Abebaw Gebeyehu**

Ethiopia

**Tesfay Gebregzabher Gebrehiwot**

Ethiopia

**Nikoletta Giatras**

UK

**Roger Gibson**

Jamaica

**Amanda Glassman**

USA

**Oliver Gruebner**

USA

**José Hagan**

USA

**Rachel Hammonds**

Belgium

**Sandya Hewamanne**

USA

**Shashivadan Hirani**

UK

**Terri Hyde**

USA

**Yulia Izati**

Indonesia

**Zarina Nahar Kabir**

Sweden

**Soumik Kalita**

India

**Mariano Kanamori Nishimura**

USA

**Godfrey Kangaude**

Malawi

**Kristi Kenyon**

Canada

**Kaveh Khoshnood**

USA

**Vincent Z. Kuuire**

Canada

**Yiannis Kyratsis**

UK

**Greg Layther**

UK

**Kenneth L Leonard**

USA

**Leslie London**

South Africa

**Svjetlana Lozo**

USA

**David Lubogo**

Uganda

**Roger Magnusson**

Australia

**Mats Malqvist**

Sweden

**Lochner Marais**

South Africa

**Maria Rosario Martins**

Portugal

**Peter Ebongue Mbondji**

Cameroon

**Gitau Mburu**

UK

**Margaret Mcconnell**

USA

**Anita Mcgahan**

Canada

**Matthew Mckenna**

USA

**Benjamin Meier**

USA

**Mohamed Izham Mohamed Ibrahim**

Malaysia

**Jose Moraes**

Brazil

**Alejandro Moreno**

USA

**Alexandra Muller**

South Africa

**Haruka Nakada**

Japan

**Brett Nelson**

USA

**Elizabeth Newnham**

Australia

**Benjamin Nganda**

Kenya

**Justice Nonvignon**

Ghana

**Noah Novogrodsky**

USA

**Jerry Okal**

Kenya

**Gorik Ooms**

Belgium

**Kwaku Oppong Asante**

Ghana

**Maria José Osis**

Brazil

**Rene Osorio**

USA

**Yin Paradies**

Australia

**Lauren Paremoer**

South Africa

**Parveen Parmar**

USA

**Robert Peck**

Tanzania

**Nasheeta Peer**

South Africa

**Emma Plugge**

UK

**Miriam Potocky**

USA

**Lisa Reynolds**

UK

**Adam Richards**

USA

**Esther Richards**

UK

**Richard Rinehart**

USA

**Giuliano Russo**

Portugal

**Elizeus Rutebemberwa**

Uganda

**Ricardo Rüttimann**

Argentina

**Shepherd Shamu**

Zimbabwe

**Geordan Shannon**

UK

**Mukta Sharma**

Thailand

**Kenneth Sherr**

USA

**Sara Shuman**

USA

**Teresa Spadea**

Italy

**Annie Sparrow**

USA

**Allison Squires**

USA

**Sarah Jane Steele**

Canada

**Alistair Stewart**

New Zealand

**Miriam Taegtmeyer**

UK

**Augustinus Ten Asbroek**

UK

**Mikiko Terashima**

Canada

**Lil Tonmyr**

Canada

**Lyndal Trevena**

Australia

**Alexander Tsai**

USA

**Remco Van De Pas**

Belgium

**Johannes Van Rooyen**

South Africa

**Murali Dhar Vemuir**

India

**Sridhar Venkatapuram**

UK

**Gwenaelle Vidal-Trecan**

France

**Kerri Viney**

Australia

**Abdul Wajid**

USA

**Joyce Wamoyi**

Tanzania

**Daniel Edward Wight**

UK

**Erin Wilson**

USA

**William Wolfe**

USA

**Junqing Wu**

China

**Qingwen Xu**

USA

**Katherine Yun**

USA

**Cathy Zimmerman**

UK

